# Towards epigenetically informed and biobank-driven precision immunotherapy in melanoma

**DOI:** 10.3389/fimmu.2026.1932718

**Published:** 2026-10-06

**Authors:** Ludovica Ciuffreda, Italia Falcone, Cristina Sorino, Alessandro Valenti, Celeste Accetta, Francesca De Nicola, Valentina Roglia, Maurizio Fanciulli, Giovanni Blandino, Edoardo Pescarmona, Simona Di Martino

**Affiliations:** 1 Gene expression and Cancer Model Unit, Department of Research, Advanced Diagnostics and Technological Innovation, Translational Research Area, IRCCS Regina Elena National Cancer Institute, Rome, Italy; 2 Radiology and Diagnostic Imaging Unit, Department of Clinical and Dermatological Research, San Gallicano Dermatological Institute IRCCS, Rome, Italy; 3 Department of Pathology Unit, Tissue Biobank, IRCCS Regina Elena National Cancer Institute, Rome, Italy; 4 Gynecologic Oncology Unit, Department of Experimental Clinical Oncology, IRCCS Regina Elena National Cancer Institute, Rome, Italy; 5 Scientific Direction, IRCCS Regina Elena National Cancer Institute, Rome, Italy

**Keywords:** biobanking, biomarker discovery, epigenomic profiling, immunotherapy, melanoma, multilayer omics, tumor microenvironment

## Abstract

Cutaneous melanoma (CM) is a highly immunogenic and therapeutically intricate solid tumor, exemplifying the interactions between tumors and the immune system. Despite the significant enhancement in survival rates for patients with advanced melanoma due to immune checkpoint inhibitors (ICIs), numerous patients either exhibit resistance to these agents or develop immune evasion mechanisms. This underscores the pressing necessity to identify biological determinants of drug responses. A significant body of research indicates that epigenetic alterations, including DNA methylation, histone modifications, and ATP-dependent chromatin remodeling, are essential in modulating melanoma immunogenicity. This encompasses the modulation of antigen presentation, innate immune detection, interferon (IFN) signaling, melanoma plasticity, and the reconfiguration of the immune microenvironment. Furthermore, in contrast to genetic mutations, these mechanisms are reversible, positioning melanoma as a prime example of epigenetically regulated and potentially therapeutically targeted immune resistance. This review examines the novel insight that epigenetic therapies can enhance the immunological activity of “cold” or immune-excluded melanomas, transforming them into inflamed, drug-responsive tumors. This is achievable by restoring antigenicity and reactivating IFN-related pathways, leading to enhanced immune cell infiltration. The paper also addresses the necessity of longitudinal biobanking combined with integrated multi-omics analyses of epigenomics, transcriptomics, proteomics, and spatial profiling to investigate dynamic tumor–immune configurations, support the validation of candidate biomarkers, and explore biologically informed patient stratification. In conclusion, we propose an integrative model connecting epigenetic vulnerability, immune reconfiguration, and biobank-driven systems biology. This model is proposed as a research framework for investigating future biomarker informed immunotherapy strategies in melanoma.

## 1 Introduction

Cutaneous melanoma (CM) is one of the most aggressive forms of skin cancer. It remains a major global health problem because of its high metastatic potential and increasing incidence, especially among populations with fair skin who are chronically exposed to ultraviolet (UV) radiation (1–3). According to the 2022 GLOBOCAN estimates, there were more than 330,000 new melanoma cases, and approximately 58,000 melanoma-related deaths occurred worldwide, highlighting the disease’s clinical relevance (4). UV radiation is the predominant etiological risk factor, inducing DNA damage and contributing to the exceptionally high tumor mutational burden (TMB) characteristic of this malignancy (5, 6). The high TMB of melanoma increases the probability of generating a broad range of potential tumor specific neoantigens, which contributes to its immunogenicity and makes melanoma a paradigmatic model for studying tumor immune interactions (7–9).

Melanoma has indeed played a central role in the development of modern cancer immunotherapy, particularly using immune checkpoint inhibitors (ICIs) including anti-Cytotoxic T-Lymphocyte Antigen 4 (CTLA-4) and anti-Programmed cell death protein 1 (PD-1). These antibodies have significantly improved long-term survival rates in subsets of patients with advanced disease (10–13). In parallel, targeted therapies directed against Mitogen-Activated Protein Kinase (MAPK) pathway alterations, particularly combined BRAF and MEK inhibition, have expanded treatment options for patients with BRAF V600 mutant melanoma (14–16). Nevertheless, melanoma exhibits significant biological heterogeneity, and durable clinical responses are limited to a subset of patients. Furthermore, even patients with highly immunogenic tumors may frequently develop primary or acquired resistance during treatment. This apparent paradox raises a fundamental biological question: why do some melanomas evade immune recognition and fail to respond to ICIs despite their high immunogenic potential? Accumulating evidence indicates that resistance cannot be fully explained by genetic alterations alone but instead emerges from a dynamic crosstalk between tumor evolution, immune pressure, and epigenetic plasticity (17–19). These processes converge on key biological programs, including impaired antigen presentation, interferon (IFN) signaling defects, phenotypic switching, and tumor immune microenvironment (TIME) remodeling, collectively driving immune escape and treatment failure (20, 21). Within this context, epigenetic regulation has emerged as one of the determinants of melanoma immune biology. DNA methylation, histone modifications, and adenosine triphosphate (ATP) dependent chromatin remodeling can reversibly modulate tumor immunogenicity, innate immune sensing, immune checkpoint regulation, and cellular plasticity, potentially affecting responses to immunotherapy (19). In contrast to fixed genomic alterations, epigenetic modifications are potentially reversible, making them putative targets for overcoming immune resistance (22, 23). Because tumor epigenetic and immune profiles may evolve during treatment, longitudinal biospecimen collection can help capture dynamic changes in tumor biology and treatment response that may not be evident in a single pre-treatment biopsy. To meet this need, a framework that integrates a comprehensive biobanking workflow with multi-omics profiling, encompassing epigenomic, transcriptomic, proteomic, and spatial analyses, will be essential to characterize dynamic tumor–immune states, identify predictive biomarkers, and inform biologically grounded therapeutic strategies (24). This critical narrative review examines the epigenetic mechanisms driving immune escape and immunotherapy resistance in melanoma. Melanoma-specific data are presented separately from findings extrapolated from other cancer types, with mechanistic and preclinical observations are distinctly separated from early clinical evidence. A hypothesis-generating framework of dynamic, epigenetically regulated immune states is proposed, with the framework itself being derived from a heterogeneous literature concerning tumors and the immune system. These proposed states are conceptualized as potentially overlapping, continuous, and multicompartmental configurations. However, their temporal dynamics, assay based definitions, and clinical relevance remain to be systematically validated. Finally, we discuss how longitudinal biobanking and integrated multi-omics approaches may support such validation efforts and inform future biomarker development, while recognizing that their clinical utility has yet to be established.

## 2 Literature search strategy

This critical narrative review was informed by searches of PubMed, Scopus, and Web of Science for English-language articles published by September 2026. Combinations of the following search terms were used: “melanoma”, “epigenetics”, “DNA methylation”, “histone modifications”, “chromatin remodeling”, “immune escape”, “immunotherapy resistance”, “biomarkers”, “multi-omics”, and “biobanking”. Priority was given to primary studies and clinical trials specific to melanoma, as well as relevant reviews. Conference abstracts and evidence from other tumor types were included only when melanoma-specific data were limited, and they are explicitly identified as preliminary or indirect evidence.

## 3 The genetic and immunological profile of melanoma

Few solid tumors show the genetic and immunological complexity of CM, which is characterized by a high TMB, significant cellular plasticity, and active remodeling of the TIME, all of which influence tumor progression and therapeutic response. At the genomic level, melanoma is predominantly driven by activating alterations in the MAPK pathway, including recurrent mutations in BRAF (~40–50% of CM melanomas), NRAS (~15–25%), and NF1 (~10–15% of cases) genes. Less frequent but biologically and clinically relevant alterations affecting KIT, Phosphatase and TENsin homolog (PTEN), Cyclin-Dependent Kinase Inhibitor 2A (CDKN2A), and genes regulating telomere maintenance (25–28).

UV radiation exerts a significant mutagenic pressure on cutaneous melanocytes and contributes to the high TMB characteristic of melanoma. The high TMB and the expanded repertoire of potential tumor specific neoantigens provide the mechanistic basis for the exceptional sensitivity of melanoma to ICIs (8, 9). However, this relationship is probabilistic rather than deterministic. Indeed, a somatic mutation only becomes immunologically visible if the mutated allele is transcribed and translated, and the resulting peptide must be proteasome processed and stably loaded onto major histocompatibility complex (MHC) class I molecules. Finally, it must be displayed at the cell surface and engaged by a T cell clone that has escaped tolerance. Therefore, TMB and predicted neoantigen load quantify potential antigenicity rather than realized antigenicity. Because most of these steps are dynamically regulated at the transcriptional and chromatin levels, epigenetic control can predict whether a mutationally rich melanoma will be immunologically visible (8).

Despite the immunogenicity potential of ICIs, durable responses remain limited to only a subset of patients. This phenomenon demonstrates the multifactorial nature of melanoma immune resistance. Tumor intrinsic mechanisms, including impaired antigen presentation, IFN signaling defects, and oncogenic pathway activation that suppresses immune infiltration, can profoundly compromise immune recognition and anti-tumor immunity (17). At the same time, tumor extrinsic components of the TIME, including immunosuppressive myeloid populations, regulatory T cells, dysfunctional stromal interactions, and inhibitory cytokine networks, further restrict effective immune responses and facilitate therapeutic evasion (20).

The number, activation status, and location of immune cells inside the TIME influence how well a patient responds to therapy. In general, melanomas that are heavily infiltrated by CD8+ T cells tend to respond better to immune checkpoint blockade, while metastatic lesions with little T cell infiltration usually have worse clinical outcomes. However, the melanoma immune microenvironment is highly heterogeneous and changes over time, shaped by the tumor’s genotype, the state of its cells, prior treatments, interactions with the stroma, and ongoing immune selection. This inherent complexity makes it implausible that a single biomarker will provide a comprehensive overview.

Accordingly, melanoma immune responses should not be interpreted as the result of isolated molecular events but as the result of dynamic interactions among genomic alterations, epigenetic plasticity, and immune ecosystem remodeling. Epigenetic regulation represents one component of this interconnected system. DNA methylation, histone modifications, and ATP-dependent chromatin remodeling can influence antigen presentation, IFN signaling, immune-checkpoint expression, and lineage-specific transcriptional programs, thereby contributing to melanoma plasticity and immune evasion (18, 22). These mechanisms and the evidence supporting their involvement in melanoma immune resistance are examined in the following sections.

## 4 The epigenetic mechanisms driving immune escape in melanoma

Epigenetic regulation helps melanoma cells evade the immune system by rewiring gene expression without altering the DNA sequence itself. Mechanisms such as DNA methylation, histone modifications, and ATP-dependent chromatin remodeling can change how visible tumor cells are to the immune system, modulate interferon signaling, alter immune checkpoint expression, transform cell identity, and increase cellular plasticity. In addition, epigenetic programs in immune and stromal cells can further shape T cell differentiation and dysfunction, drive myeloid cell polarization, affect antigen-presenting cell function, and remodel the tumor immune microenvironment. These processes are interconnected and may coexist within the same tumor, differ across regions and cell types, or vary depending on the stage of treatment. Although some epigenetic changes can be reversed in experimental settings, this does not automatically translate into clinically useful therapies. Most available epigenetic drugs lack the precision to act on specific genes or cell types, and they can have variable, sometimes opposing, effects on tumor versus immune cells. For this reason, the following sections distinguish mechanistic insights from therapeutic evidence and clarify whether the findings originate from melanoma-specific preclinical models, patient samples, clinical studies, or data extrapolated from other cancers.

### 4.1 DNA methylation and tumor cell immunogenicity

Aberrant DNA methylation at CpG dinucleotides within gene promoters and enhancers is a hallmark of cancer and one of the most extensively characterized epigenetic mechanisms driving immune evasion in melanoma. In this context, DNA hyper- and hypomethylation events are not randomly distributed but affect specific gene categories involved in tumor progression and immune escape. Notably, genome-wide methylation analyses of 50 metastatic melanomas, validated in 242 primary and metastatic tumors from The Cancer Genome Atlas, identified three distinct methylation subgroups that were independent of BRAF and NRAS mutational status. They showed a range of phenotypes from proliferative to immune-infiltrated (29). The first subgroup was characterized by hypermethylation of specific promoters, demethylation of repetitive elements, high tumor purity, frequent deletion of a critical gene, and upregulation of cell cycle regulators. This subgroup also exhibited features of a proliferative transcriptional phenotype, as did the second subgroup, whereas the third retained unmethylated promoters and displayed higher immune and stromal infiltration. MS1 was associated with shorter survival in the TCGA metastatic group, supporting the idea that melanomas follow either a proliferative or immune-microenvironment trajectory that is not captured by driver mutation profiling (29). Promoter hypermethylation of multiple tumor suppressor genes drives the dysregulation of key biological processes associated with melanoma progression. Among these genes, the epigenetic silencing of the tumor suppressor PTEN through promoter hypermethylation has been extensively documented in melanoma and associated with activation of PI3K–AKT signaling and unfavorable clinical features or outcomes (30–32). However, the available evidence primarily supports a role for PTEN methylation in melanoma progression and prognosis; its direct contribution to immune escape has not been established by these studies. Aberrant DNA methylation can impair a tumor’s ability to evade the immune system by reducing or abolishing its capacity to present antigens via MHC class I molecules. In their review, Dhatchinamoorthy et al. summarize mechanisms of immune evasion mediated by loss of MHC class I antigen presentation across multiple tumor types, highlighting that these mechanisms are, in part, tumor specific. In melanoma, they describe genetic alterations, including mutations and deletions affecting MHC-I, β2-microglobulin, TAP, and tapasin, as well as defects in the JAK/STAT pathway, together with epigenetic mechanisms. Specifically, hypermethylation of promoters or enhancers regulating genes encoding MHC-I and antigen-processing machinery components, increased repressive histone marks (H3K27me3) mediated by the PRC2/EZH2 complex, and reduced expression of transcription factors such as NLRC5 and IRF1 have all been reported (33). Collectively, these alterations are associated with poor prognosis and resistance to immune checkpoint inhibitors.

The cGAS–STING pathway enhances antitumor immunity by stimulating a type I interferon response and increasing MHC class I expression, which amplifies tumor antigenicity. In melanoma, however, this pathway is often suppressed by an epigenetic rather than genetic mechanism. Falahat et al. demonstrated that promoter hypermethylation of the cGAS–STING pathway epigenetically suppresses tumor intrinsic innate immune signaling in melanoma, reducing tumor antigenicity and impairing T cell recognition. Pharmacologic DNA inhibition of DNA methyltransferases with 5-aza-2′-deoxycytidine restored STING signaling, increased MHC-I expression, enhanced IFN-γ release by tumor infiltrating lymphocytes, and promoted greater cytotoxic T cell mediated lysis of melanoma cells. These findings suggest that STING promoter methylation may represent a candidate molecular readout of impaired innate immune sensing and provide a preclinical rationale for investigating DNA methyltransferase inhibitors in combination with T cell based immunotherapies. However, its potential value for patient stratification or treatment selection remains hypothetical and requires prospective validation in clinically annotated melanoma cohorts (23).

In addition, CARD domain-containing 5 (NLRC5) has been identified as a critical regulator of antitumor immunity in human neoplasia (including melanoma, cervical, bladder, rectal, and head and neck cancers). NLRC5 acts as a master transcriptional activator of the MHC class I antigen presentation machinery, regulating the expression of genes vital for tumor recognition by CD8+ T cells. Its expression and activity are frequently reduced by promoter methylation, copy number loss, and somatic mutation. This results in defective MHC class I presentation, impaired antitumor immune responses, and poorer patient prognosis. Like STING, NLRC5 promoter methylation is a recurrent node underlying tumor immune escape (34). Interestingly, an integrative bioinformatic analysis of 461 cutaneous melanoma patients from TCGA, validated across multiple independent GEO cohorts, and in patients treated with anti-CTLA-4, anti-PD-1, or adoptive T cell therapy, showed a lower NLRC5 expression due to promoter hypermethylation. This was associated with reduced CD8+ T cell infiltration, impaired antitumor immune activity, a worse prognosis, and decreased responsiveness to ICIs. This suggests that NLRC5 may play a role in immune evasion and as a biomarker for immunotherapy response (34, 35). Consistently, restoring NLRC5 expression through targeted demethylation of its promoter using a CRISPR/dCas9-based epigenetic editing system reactivated MHC class I antigen presentation in murine melanoma and human breast cancer cells, enhanced CD8+ T cell activation, and suppressed tumor growth. It also potentiated anti-PD-1 blockade in immunocompetent mice. These findings support the view that suppression of the NLRC5 axis through methylation is a mechanism of melanoma immune escape that can be targeted therapeutically (36).

Numerous studies have shown that DNA methylation plays a role in melanoma immunogenicity by regulating cancer testis antigens (CTAs), a highly immunogenic class of tumor associated antigens that are normally restricted to germ cells of the testis and placenta. However, they are aberrantly re-expressed in cancer. The expression of CTA families such as melanoma-associated antigen-A (MAGE-A), New York esophageal squamous cell carcinoma 1 (NY-ESO-1), G antigen (GAGE), and synovial sarcoma X breakpoint (SSX) can elicit humoral and T cell mediated immune responses (37). Preclinical studies indicate that their expression is influenced by promoter methylation, with hypomethylation permitting transcriptional reactivation, and show that DNA hypomethylating agents, including 5-aza-2′-deoxycytidine, can induce or enhance CTA expression in melanoma cells. This pharmacological inducibility provides a rationale for combining hypomethylating agents with CTA-directed immunotherapy to upregulate target antigens in tumors where their expression is absent or intratumorally heterogeneous (38, 39).

### 4.2 Histone modifications influencing the tumor microenvironment

Beyond DNA methylation, histone modifications are a critical epigenetic mechanism that regulates melanoma progression and TIME remodeling. By modulating chromatin accessibility and transcriptional activity, histone methylation and acetylation influence melanoma cell plasticity, metastatic potential, immune interactions, and therapeutic response. There is increasing evidence that dysregulation of histone-modifying enzymes may contribute to melanoma heterogeneity and immune escape, although the resulting effects depend on the specific enzyme involved, the cellular compartment, and the biological context.

Trimethylation of lysine 27 on histone H3 (H3K27me3) is one of the most extensively studied repressive histone modifications in melanoma. This modification is catalyzed by Enhancer of Zeste Homolog 2 (EZH2), a histone methyltransferase that forms the catalytic core of Polycomb Repressive Complex 2 (PRC2), which silences transcription by promoting chromatin compaction at target promoters. Analysis of TCGA data suggests that approximately 20% of CM patients have EZH2 genomic amplification, EZH2 protein overexpression, the activating mutation Y646, or alterations in PRC2 components, all of which are associated with lower survival rates. Mechanistically, EZH2-driven H3K27me3 deposition represses tumor suppressor networks that control proliferation and restrain invasion, thereby sustaining melanoma growth and metastatic dissemination. In preclinical melanoma models, genetic or pharmacological inhibition of EZH2 impairs tumor cell proliferation and reactivates selected tumor suppressive transcriptional programs. However, the available evidence supporting these tumor intrinsic effects remains predominantly preclinical (40).

In addition to these tumor intrinsic effects, EZH2 can regulate tumor cell intrinsic immune-evasion programs. The relationship between histone modifications and immune checkpoint regulation in melanoma is highly dynamic and context dependent. In murine melanoma models, anti-CTLA-4 or interleukin-2 (IL-2) treatment induced intratumoral T cell accumulation and TNF-α production, which increased EZH2 expression in melanoma cells. Increased EZH2 activity was associated with H3K27me3-dependent repression of genes involved in antigen presentation and immune-cell recruitment, including MHC class I genes and the chemokines CXCL9 and CXCL10. Genetic inactivation or pharmacological inhibition of EZH2 with GSK503 partially reversed this adaptive program and improved the antitumor activity of anti-CTLA-4 or IL-2 treatment in preclinical melanoma models (41). These findings identify EZH2 as a mediator of adaptive resistance under experimental conditions, but they do not establish the efficacy of EZH2 inhibition in patients receiving immunotherapy (41).

A distinct EZH2-dependent mechanism of immune escape involves H3K27me3-mediated repression of STING expression in melanoma cells. Reduced STING signaling activity attenuates type I interferon responses and decreases recruitment and activation of antitumor immune cells, thereby contributing to an immune-poor, or “cold” tumor phenotype (42, 43). Consistent with this mechanism, Xu et al. showed that EZH2 inhibition increased STING expression in melanoma cell lines and enhanced the antitumor activity of a STING agonist in murine models. Compared with either intervention alone, combined EZH2 inhibition and STING activation enhanced immune-related signaling, increased immune cell infiltration, and improved tumor control (44). These findings provide preclinical evidence of a functional interaction between EZH2-dependent chromatin repression and tumor cell-intrinsic STING signaling. However, this therapeutic combination has not been clinically evaluated in melanoma, and its efficacy and safety in patients remain unknown.

Histone acetylation provides an additional mechanism for immune regulation. Histone deacetylases (HDACs) remove acetyl groups from histone and nonhistone proteins, may produce different effects that vary according to HDAC class, substrate, and cellular context. Recent evidence has shown that, in melanoma cell models, inhibition of Class I HDACs increases histone acetylation at the CD274 promoter and upregulates PD-L1 and PD-L2 expression. This reveals an important potential therapeutic paradox: HDAC inhibition enhances tumor immunogenicity and sensitizes melanoma to immune checkpoint blockade, but it may also promote adaptive resistance by inducing PD-L1, supporting the rationale for combining HDAC inhibition with checkpoint-targeted therapies. These apparently divergent effects indicate that HDAC inhibition should not be interpreted as uniformly immunostimulatory or immunosuppressive, underscoring the importance of carefully evaluating drug class, dose, sequence, and cellular context (45, 46).

In line with the concept of these enzymes having a broader immunoregulatory role, a recent computational study integrating publicly available melanoma datasets reported that elevated HDAC4 expression has been linked to reduced T-cellinflamed gene expression signatures. Lower estimated CD8+ T cell infiltration, increased methylation of selected immune-related genes, and less favorable outcomes in patients receiving ICIs (47). These findings suggest that HDAC4 may be associated with an immune-excluded phenotype, but they do not demonstrate that it directly causes immune exclusion or that it is a reliable biomarker for ICI response. Taken together, these studies indicate that histone-modifying enzymes can influence tumor cell transcriptional programs, antigen presentation, inflammatory signaling, and immune-checkpoint expression in melanoma. However, most mechanistic and therapeutic evidence comes from cell-based experiments, murine models, or retrospective analyses of public datasets. The clinical value of targeting EZH2 or HDACs to overcome immunotherapy resistance, therefore, remains to be determined in prospective melanoma studies.

### 4.3 Chromatin remodeling and immune editing

In addition to DNA methylation and histone modifications, ATP-dependent chromatin remodeling contributes to melanoma immunogenicity and tumor immune interactions. The SWI/SNF (SWItch/Sucrose Non-Fermentable) complex functions as a master regulator of chromatin accessibility, increasing DNA accessibility for transcription factors and other regulators. In melanoma, recurrent alterations affecting SWI/SNF components, particularly the AT-rich interaction domain (ARID) proteins ARID1A, ARID1B, and ARID2, are among the most frequent mutations involving epigenetic regulation (48). However, ARID2 is a core component of the polybromo-associated BAF (PBAF) complex. Experimental evidence suggests that disrupting PBAF components can modify accessibility at IFN-responsive loci, alter inflammatory transcriptional responses, and alter susceptibility to T cell mediated cytotoxicity. The effects of SWI/SNF alterations on the immune system depend on the subunit and the context (48).

Chromatin remodeling also intersects with melanoma immune evasion by regulating tumor cell plasticity. Melanoma cells exhibit remarkable epigenetic adaptability and can transition between differentiated melanocytic states and less differentiated, invasive states in response to environmental and treatment-related signals. Sævarsson and collaborators have shown that decreased activity of microphthalmia-associated transcription factor (MITF), a central regulator of melanocytic lineage identity, is associated with reduced accessibility at melanocytic differentiation loci. They also found increased accessibility at regulatory regions associated with inflammatory signaling, migration, and immune adaptation (49).

Using Assay for Transposase-Accessible Chromatin (ATAC)-seq analyses, Sævarsson et al. demonstrate that IFN-γ-induced chromatin remodeling is markedly amplified in melanoma cells model where MITF is low (siRNA-mediated MITF depletion). MITF-depleted cells exhibited an enhanced chromatin response to IFN-γ, including increased accessibility at inflammatory and IFN-responsive regulatory regions and enrichment of interferon regulatory factor (IRF) and signal transducer and activator of transcription (STAT) motifs. These changes were accompanied by reduced accessibility at loci associated with melanocytic differentiation and increased expression of inflammatory mediators and PD-L1 (49, 50).

Notably, chromatin accessibility works with DNA methylation and histone modifications to create a coordinated epigenetic program of immune evasion. This supports the idea of combining epigenetic therapies with immune checkpoint blockade to overcome adaptive resistance in melanoma. However, these approaches remain experimental, and their efficacy, specificity, and effects across tumor and immune compartments require further investigation.

### 4.4 Phenotype switching as an epigenetically regulated mechanism of immune adaptation

Melanoma progression and therapy resistance may be influenced by non-mutational mechanisms, such as phenotype switching. In this process, cells can reversibly transition between a differentiated and an undifferentiated state in response to environmental, immune, and therapeutic pressures. This plasticity, which is mediated by epigenetic and transcriptional reprogramming rather than genetic selection, allows cells to rapidly adapt to a shifting microenvironment (50–52).

Early models described two main melanoma phenotypes: an MITF-high melanocytic/proliferative state, associated with melanocytic differentiation and proliferation, and an MITF-low/AXL-high invasive state, associated with reduced differentiation, increased migration, inflammatory signaling, and resistance to therapy (53, 54). However, transcriptomic analysis reveals that melanoma plasticity exists along a gradient of intermediate differentiation states rather than in distinct phenotypic groups. This finding further supports the idea that dedifferentiation occurs gradually through changes in the transcriptome (55). Consistent with this model, single-cell RNA sequencing of BRAF-mutant melanoma during combined RAF and MEK inhibition identified multiple drug-tolerant transcriptional states that coexisted within minimal residual disease in patient-derived xenografts and patient biopsies. These included a neural-crest stem cell–like state regulated in part by retinoid X receptor gamma (RXRG) (51). Thus, the available evidence supports a continuum of potentially coexisting and context-dependent cellular states.

At the molecular level, phenotype switching is regulated by complex networks involving lineage-specific transcription factors, changes in chromatin accessibility, and epigenetic modifications. MITF, and SOX10 regulate of melanocytic identity, activates differentiation-associated programs. In contrast, dedifferentiated states are characterized by the activation of invasive regulators, including AXL, BRN2, Yes-Associated Protein (YAP)/Transcriptional co-activator with PDZ-binding motif (TAZ), Zinc finger E-Box-binding homeobox (ZEB)1, and the Activator Protein (AP)-1 family of transcription factors. There is also extensive remodeling of the chromatin of immune-regulatory and inflammatory loci (52). Notably, ATAC-seq studies have revealed that treatment pressure results in significant changes in chromatin accessibility. These changes include the disappearance of SOX10-dependent melanocytic programs and the emergence of Transcriptional Enhanced Associate Domain (TEAD)/AP-1-driven transcriptional states linked to adaptive resistance. Functional perturbation of TEAD factors reduced expression of the invasive gene program, supporting their contribution to this cellular state (56). These findings connect phenotype switching with changes in the regulatory chromatin landscape, although the precise epigenetic mechanisms and their reversibility may differ across models and treatment contexts.

Additionally, microenvironmental signals help to maintain this plasticity. Inflammatory cytokines, such as IFN-γ, Tumor Necrosis Factor (TNF)-α, and Transforming Growth Factor (TGF)-β, can promote dedifferentiation by altering the transcriptional and chromatin landscapes, leading to immune-adaptive phenotypes characterized by reduced antigenicity and increased immune tolerance (57). This study provides direct evidence that inflammatory pressure can select or induce an immune-evasive, differentiated phenotype. Nevertheless, individual cytokines should not be assumed to produce identical effects: their consequences depend on signaling duration, cellular state, and the composition of the tumor microenvironment.

Non-canonical Wingless-type MMTV integration site family, member 5A (Wnt5A) signaling, may also contribute to phenotype switching. Under hypoxic conditions, increased WNT5A/ROR2 signaling was associated with suppression of melanocytic differentiation programs, acquisition of a more invasive phenotype, and reduced sensitivity to BRAF inhibition. Experimental modulation of the ROR1–ROR2 switch supported a functional contribution of this pathway to hypoxia-induced plasticity and targeted-therapy resistance (58).

From a translational perspective, phenotype switching serves as both a mechanism of cellular plasticity and a clinically relevant epigenetic immune adaptation program that links tumor dedifferentiation to therapeutic evasion. This continuous process may partly explain why some melanomas initially respond to immune checkpoint blockade but later relapse, even when tumor mutational burden remains intact, and baseline immunogenicity appears to be present. Therefore, treatment methods that prevent or reverse dedifferentiation, such as epigenetic priming approaches focusing on chromatin remodeling and transcriptional plasticity, may provide opportunities to regain long-term susceptibility to immunotherapy and targeted treatments.

## 5 Epigenetic reprogramming to overcome immunotherapy resistance

Despite the clinical success of ICIs, only some patients with melanoma experience prolonged clinical benefits, while others show either primary resistance or later develop adaptive immune escape during therapy. This variability reflects the biological heterogeneity of melanoma and the dynamic interactions among tumor cells, immune populations, stromal components, and treatment-induced selective pressures. Increasing evidence indicates that immune resistance in melanoma is driven not only by permanent genetic changes but also by potentially reversible and context-dependent epigenetic programs that can shape tumor immunogenicity. These programs support immune evasion by fostering T cell exclusion, weakening IFN responses, and maintaining dysfunctional immune states (59, 60). The reversible nature of these epigenetic alterations has generated interest in epigenetic priming, where epigenetic therapies are used primarily as immune sensitizers to restore tumor immunogenicity rather than as tumor killing agents. Preclinical findings suggest that such interventions may increase tumor antigenicity or modify selected immune programs. However, epigenetic reversibility should not be equated with established therapeutic actionability because currently available agents generally lack locus and cell-type specificity and may exert divergent effects in tumor and immune compartments.

### 5.1 Preclinical epigenetic strategies for immune sensitization in melanoma

DNA methyltransferase inhibitors (DNMTis), such as azacitidine and decitabine, are among the most extensively studied epigenetic strategies for immune sensitization in melanoma. Their therapeutic rationale relies on complementary immunological mechanisms that enhance tumor recognition, primarily by restoring epigenetically silenced tumor antigens. DNMT inhibition has been shown to promote the re-expression of cancer-testis antigens (CTAs), including members of the melanoma-associated antigen A (MAGE-A) family and New York esophageal squamous cell carcinoma 1 (NY-ESO-1), which are frequently regulated through promoter methylation. Increased antigen expression may enhance tumor cell recognition, although antigen re-expression alone does not demonstrate effective antitumor immunity or clinical responsiveness to ICIs. Another important mechanism is the induction of viral mimicry, a process in which DNMT inhibition demethylates endogenous retroviral elements (ERVs), promoting the transcription of retroviral elements and the accumulation of double-stranded RNA (dsRNA). This accumulation triggers innate antiviral sensing pathways, including Toll-Like Receptor 3 (TLR3) and Mitochondrial Antiviral-Signaling protein (MAVS) signaling, resulting in the activation of type INFs and stronger inflammatory immune programs. In a landmark study, Chiappinelli et al. showed that DNMT inhibition initiates a viral defense transcriptional program that sensitizes tumors to ICIs blockade and correlates with durable responses in melanoma patients treated with anti-CTLA-4 therapy (61). However, this association does not establish the signature as a prospectively validated predictive biomarker in melanoma. In addition to promoting IFN signaling, DNMTis help remodel the tumor microenvironment. Preclinical studies suggest that DNMT inhibition can remodel immune ecosystems by increasing IL-33 expression through demethylation of a regulatory region within the *IL33* gene. Which ultimately improves responsiveness to PD-1 blockade. Taken together, these findings present DNMT inhibition as a promising means of restoring immune competence in resistant melanoma. Decitabine-dependent activation of the IL-33/ST2 axis promoted the recruitment of T cells and eosinophils and, in combination with exogenous IL-33, enhanced the response to PD-1 blockade in melanoma-bearing mice (62) These findings provide melanoma-specific preclinical evidence that DNMT inhibition can influence tumor–immune interactions; however, the efficacy and safety of this combination have not been established in patients. Histone deacetylase inhibitors (HDACis) have a range of immunomodulatory effects that extend beyond chromatin remodeling. In melanoma, HDAC inhibition increases antigen presentation, promotes the activation and infiltration of cytotoxic CD8+ T cells, and alters suppressive immune compartments, including myeloid-derived suppressor cells (MDSCs), regulatory T cells (Tregs), and tumor associated macrophages. These effects vary according to the HDAC isoform targeted, drug selectivity, dose, treatment schedule, and cellular compartment; therefore, HDAC inhibition should not be considered uniformly immunostimulatory. HDAC inhibition has also been linked to the regulation of innate immune checkpoints. For example, selective HDAC6 inhibition can reduce CD47 expression on melanoma cells and SIRPα expression on macrophages, promoting macrophage-mediated phagocytosis and strengthening the therapeutic effect of anti-CD47 treatment (59). Woods et al. demonstrated, in experimental models, that class I HDAC inhibition increases PD-L1 expression and renders melanoma cells susceptible to PD-1/PD-L1 blockade. This provides a mechanistic rationale for combining HDAC inhibitors with ICIs in poorly inflamed melanomas (46). This concept is supported by clinical data from the ENCORE-601 phase Ib/II trial, which is discussed below (63, 64). The apparently paradoxical induction of checkpoint-ligand expression further demonstrates that the effects of HDAC inhibition are context-dependent and that treatment sequence and immune context require careful evaluation. Bromodomain and extra-terminal domain (BET) inhibitors influence tumor immunogenicity by regulating transcriptional programs that control immune evasion, inflammatory signaling, and tumor adaptation. Bet inhibition disrupts chromatin-dependent transcription by displacing bromodomain-containing protein 4 (BRD4) from acetylated histones which, in turn, suppresses the expression of immunesuppressive mediators, such as the PD-L1 gene. Hogg et al. demonstrated that BRD4 inhibition decreases constitutive and IFN-γ–induced PD-L1 expression in melanoma models, suggesting a direct epigenetic mechanism that controls immune checkpoint expression (65). Additionally, preclinical studies combining the BET inhibitor JQ1 with anti-PD-1 therapy have demonstrated synergistic antitumor effects, increased MHC class I expression, improved T cell mediated tumor recognition, and greater antitumor activity than either treatment alone (66). These observations support further investigation but do not demonstrate clinical efficacy or identify a validated biomarker for patient selection.

Combination strategies involving innate immune activation also represent an emerging therapeutic opportunity. Restoration of STING signaling and modulation of the CD47/Signal-Regulatory Protein Alpha (SIRP)α axis may together strengthen both innate and adaptive immune responses, offering complementary mechanisms to overcome immune exclusion (23, 67, 68). However, these approaches have primarily been evaluated in cell-based and animal models, and their safety, optimal sequencing, and efficacy in patients with melanoma have not been established. Finally, epigenetic therapies may cooperate with MAPK pathway inhibition, particularly in melanomas undergoing phenotype switching toward dedifferentiated MITF low/AXL high states, which are frequently associated with therapeutic resistance and immune escape. By limiting adaptive cellular plasticity, epigenetic modulation could help maintain the durability of targeted therapy responses (52, 69). Nevertheless, the capacity of these treatments to prevent or reverse phenotype switching in patients remains hypothetical. Collectively, these approaches suggest that epigenetic priming may link tumor intrinsic transcriptional reprogramming with broader remodeling of the immune ecosystem. **Figure 1** summarizes selected epigenetic alterations, their context-dependent effects within melanoma cells, downstream immune consequences, and investigational therapeutic strategies. The depicted relationships are schematic and do not imply that all alterations produce the same immune phenotype or that all interventions restore sensitivity to ICIs. Most mechanistic and combination-treatment evidence remains preclinical, while melanoma-specific clinical evidence is limited.

**Figure 1:**
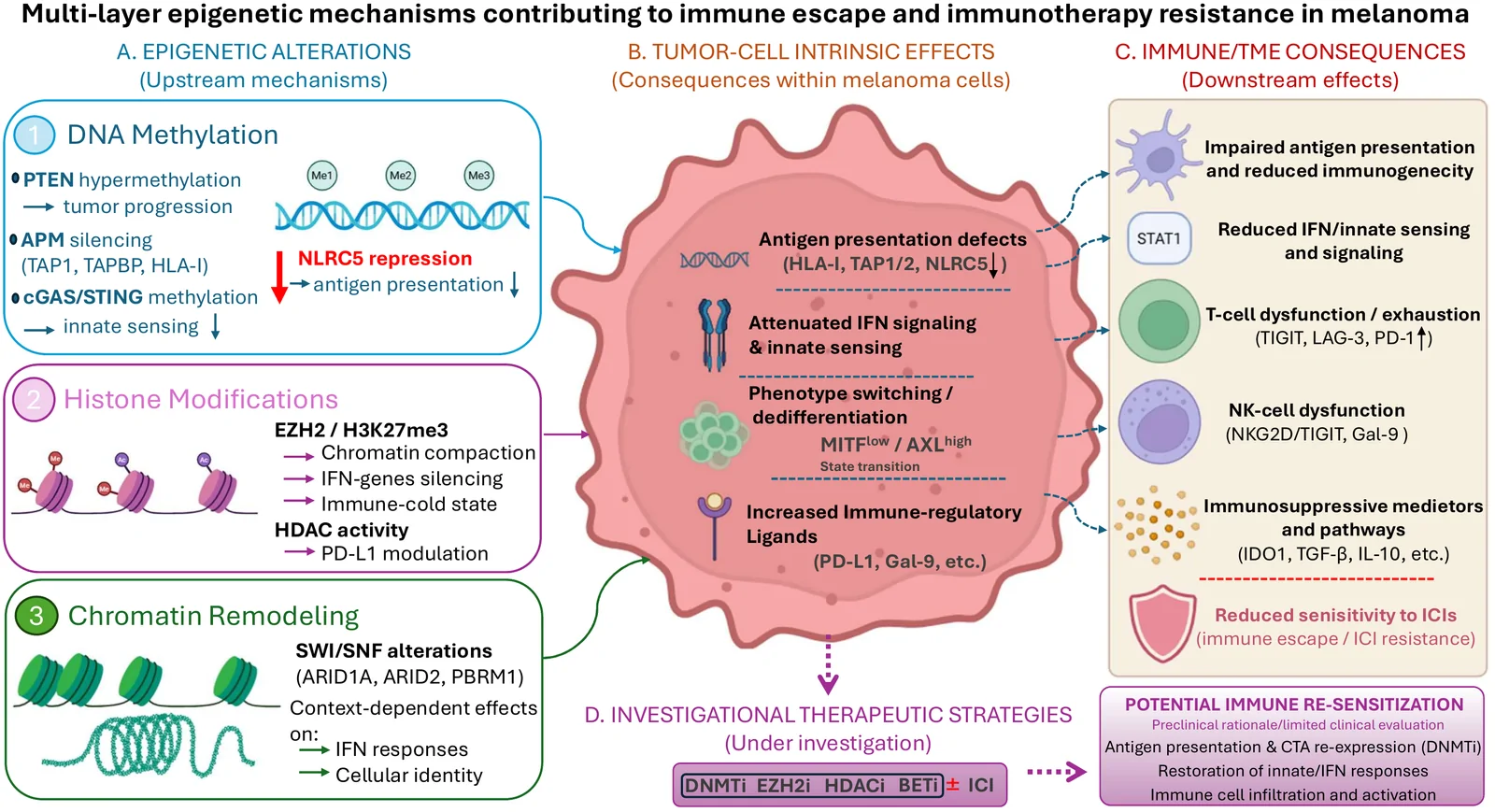
Multi-layer epigenetic mechanisms contributing to immune escape and immunotherapy resistance in melanoma. The figure summarizes the hierarchical relationship between epigenetic alterations, tumor cell intrinsic immune dysfunction, downstream immune and tumor microenvironment consequences and investigational therapeutic strategies in melanoma. **(A)** Epigenetic alterations include DNA methylation, histone modifications and chromatin-remodeling defects. DNA methylation may contribute to immune escape through PTEN hypermethylation, silencing of antigen-processing machinery, repression of NLRC5 and methylation of cGAS/STING, whereas aberrant cancer-testis antigen expression may reflect broader epigenetic dysregulation. Histone modifications, including EZH2-mediated H3K27me3 and HDAC activity, can promote chromatin compaction, suppression of IFN-responsive programs and modulation of PD-L1 expression. Loss of SWI/SNF components, including ARID1A, ARID2 and PBRM1, may alter IFN signaling and favor MITF-low/AXL-high dedifferentiated states. **(B)** Tumor cell intrinsic effects include impaired antigen presentation, attenuated IFN and innate immune sensing, phenotype switching/dedifferentiation and increased expression of immune-regulatory ligands. **(C)** Downstream immune/TME consequences include reduced tumor immunogenicity, impaired IFN/innate signaling, T cell dysfunction or exhaustion, NK-cell dysfunction, an immunosuppressive cytokine milieu and reduced sensitivity to ICIs. **(D)** Investigational therapeutic strategies include DNMT, EZH2, HDAC and BET inhibition, alone or in combination with ICIs. These approaches have a biological rationale for potential immune re-sensitization, including enhancement of antigen presentation, restoration of innate/IFN responses and increased immune-cell infiltration or activation; however, these effects are supported predominantly by preclinical or limited clinical evidence and should not be interpreted as established clinical benefit in melanoma. Solid relationships represent established or recurrently reported melanoma associated mechanisms, whereas dashed relationships indicate predominantly preclinical, proposed, or incompletely validated associations; therapeutic relationships remain investigational. APM, antigen-processing machinery; BETi, bromodomain and extraterminal domain inhibitor; CTA, cancer-testis antigen; DNMTi, DNA methyltransferase inhibitor; EZH2i, enhancer of zeste homolog 2 inhibitor; HDACi, histone deacetylase inhibitor; IFN, interferon; ICI, immune checkpoint inhibitor; NK, natural killer; PD-1, programmed cell death protein 1; PD-L1, programmed death-ligand 1; TGF-β, transforming growth factor-beta; TME, tumor microenvironment. Figure created with BioRender.com.

### 5.2 Translational and clinical landscape

Despite extensive preclinical evidence, clinical validation of epigenetic priming in melanoma remains limited. The main melanoma-specific evidence comes from ENCORE-601, a study that evaluated the class I HDAC entinostat in combination with pembrolizumab in patients exhibiting anti-PD-1 refractoriness. In the reported melanoma cohort, 53 evaluable patients were treated, and 10 objective responses were observed, corresponding to an objective response rate of approximately 19%; the reported median duration of response was 12.5 months. These findings were disseminated primarily as conference data rather than as a complete peer-reviewed melanoma-specific publication (63, 64). The single-arm design, modest sample size, absence of a pembrolizumab-alone control group, and selected anti-PD-1-experienced population preclude attribution of the observed activity specifically to epigenetic sensitization. Therefore, ENCORE-601 provides a preliminary clinical signal but does not establish that entinostat restores sensitivity to PD-1 blockade in melanoma (70). A separate cohort of ENCORE-601 evaluated entinostat plus pembrolizumab in patients with metastatic non-small-cell lung cancer who had experienced disease progression during previous anti-PD-(L)1 therapy. Although the NSCLC results were subsequently published in a peer-reviewed article, they represent cross-tumor evidence and should not be interpreted as clinical validation of epigenetic priming in melanoma (64).

Other epigenetic agents, including DNMT, EZH2, BET, and LSD1 inhibitors, remain supported predominantly by melanoma cell line studies, animal models, or clinical evidence derived from other malignancies. In particular, clinical studies of iadademstat have mainly involved acute myeloid leukemia and should not be presented as evidence of clinical activity in melanoma (71–73). At the same time, personalized neoantigen vaccination, including mRNA-4157/V940 combined with pembrolizumab in KEYNOTE-942, has become a promising addition to checkpoint inhibition. In patients with high-risk resected melanoma, the individualized mRNA-based neoantigen vaccine mRNA-4157/V940 (intismeran autogene) combined with pembrolizumab significantly improved recurrence-free survival compared with pembrolizumab alone in the randomized phase IIb KEYNOTE-942 trial. This provides the concept that precision vaccination can strengthen anti-tumor immune surveillance and maintain lasting immunologic memory (74). However, current evidence remains preliminary and insufficient to establish clinical benefit, optimal treatment sequence, or biomarker-defined patient selection. Major unresolved issues include treatment-related toxicity, tumor and immune-cell heterogeneity, narrow therapeutic windows, lack of locus and cell type specificity, and the absence of prospectively validated biomarkers.

### 5.3 Candidate epigenetic immune states as profile associated with ICI resistance

Despite the transformative clinical impact of ICIs, currently available biomarkers, including PD-L1 expression, TMB, and immune infiltration scores, only partly capture the biological complexity of melanoma and often fail to predict durable therapeutic responses. Growing evidence indicates that immune resistance arises not from isolated molecular alterations alone but from dynamic and reversible interactions among tumor evolution, immune pressure, and epigenetic plasticity (17, 18, 22).

On the basis of this heterogeneous literature, we propose “epigenetic immune states” as a hypothesis-generating framework for organizing selected patterns of melanoma immune resistance. These states should not be interpreted as discrete biological entities, a validated melanoma classification, or established prognostic or predictive biomarkers. They represent potentially overlapping, continuous, and multicompartmental configurations involving tumor cells, immune populations, and stromal components. Multiple states may coexist within the same lesion or differ across metastatic sites and treatment timepoints. For each proposed state, **Table 1** should distinguish the predominant biological compartment, candidate molecular features, potential assay or readout, temporal context, disease specificity, and level of evidence. Evidence should be classified as melanoma-specific clinical, melanoma preclinical, cross-tumor, or conceptual.

**Table 1:** Proposed epigenetic immune configurations in melanoma and candidate approaches for their investigation.

Proposed configuration	Predominant compartment	Candidate biological features	Candidate assays/readouts	Temporal context	Evidence level
Antigen-presentation-deficient	Tumor cell	Reduced HLA-I, B2M, TAP1/TAP2, TAPBP or NLRC5; genetic and/or epigenetic causes.	MHC-I protein; APM-gene RNA/protein; locus-specific methylation; antigen-specific T cell recognition.	Pretreatment or acquired; may differ across lesions.	Melanoma preclinical and melanoma-specific clinical association; not validated.
Innate immune-silenced	Tumor cell	Repressed cGAS–STING signaling with attenuated type I IFN responses and MHC-I induction.	cGAS/STING expression and methylation; downstream IFN signaling; functional response to STING activation.	Pretreatment or treatment-associated; reversibility shown experimentally.	Melanoma preclinical.
Dedifferentiated immune-adaptive	Tumor cell, with microenvironmental inputs	Reduced MITF/SOX10 and melanocytic antigens; AXL/AP-1/TEAD programs; state-specific chromatin accessibility.	Tumor cell resolved RNA/ATAC profiling; MITF, SOX10 and AXL; melanocytic-antigen protein; spatial analysis.	Dynamic under inflammatory or RAF/MEK pressure; heterogeneous and potentially coexisting states.	Melanoma preclinical and melanoma-specific patient-sample evidence.
IFN-responsive but T-cell dysfunctional	Tumor cells and infiltrating T cells	Tumor IFN response and PD-L1 with variable T cell dysfunction; progenitor-like and terminal states must be distinguished.	IFN signature and PD-L1; single-cell or multiplex T cell phenotyping (including TCF7, TOX, PD-1, TIM-3 and LAG-3); proliferation, cytokine and cytotoxicity assays.	Often treatment-associated or linked to chronic inflammation; compartment- and state-dependent.	Melanoma-specific clinical association and conceptual integration; no standardized panel.
Myeloid-dominant suppressive	Myeloid and stromal tumor microenvironment	Macrophages/MDSCs, suppressive cytokines, high myeloid-to-CD8^+^ ratio and T cell exclusion; epigenetic basis not established.	Cell-type-resolved immune profiling; cytokines; myeloid-to-CD8^+^ ratios; spatial imaging; enriched-cell chromatin or transcriptomic profiles.	Pretreatment or adaptive; strongly influenced by site and cellular composition.	Conceptual with limited melanoma-specific association; direct epigenetic evidence is limited.
Epigenetically reactivated antigenic	Tumor cell; treatment-induced	DNMT-inhibitor-associated CTA re-expression (MAGE-A, NY-ESO-1, GAGE and SSX), which does not ensure antigen processing or recognition.	CTA RNA/protein and methylation as pharmacodynamic readouts; antigen-presentation and T-cell recognition assays.	On treatment; timing- and dose-dependent; no validated therapeutic window.	Melanoma preclinical and cross-tumor clinical context; not a validated predictive state.

The configurations are hypothesis-generating, potentially overlapping, continuous and multicompartmental. They are neither validated melanoma categories nor predictive biomarkers. Evidence is classified as melanoma-specific (clinical or preclinical), cross-tumor or conceptual. Candidate readouts require longitudinal, prospective and cell-type-resolved validation, and currently do not support treatment selection.

#### 5.3.1 Antigen presentation deficient state

A first proposed tumor cell intrinsic configuration is characterized by impaired antigen processing or presentation. This state is characterized by the silencing of genes that control antigen processing and presentation, including HLA class I molecules, B2M, TAP1/TAP2, TAPBP, and their master regulator, NLRC5. Both genetic and epigenetic alterations can contribute to suppression of the antigen-presentation machinery, and promoter methylation should not be assumed to be the causal mechanism for every affected component (33–36). Reduced peptide presentation may diminish recognition by cytotoxic T lymphocytes and contribute to immune escape. Candidate readouts include MHC class I protein expression, expression of antigen-processing genes, promoter or enhancer methylation, and functional assessment of antigen-specific T cell recognition. However, no standardized combination of these measurements currently defines a clinically validated antigen-presentation-deficient state. This configuration may help explain why some melanomas fail to respond despite a high TMB, but it cannot presently identify individual patients who are unlikely to benefit from ICIs or who should receive epigenetic priming.

#### 5.3.2 Innate immune silenced state

A second proposed tumor cell-intrinsic configuration involves suppression of innate immune sensing through epigenetic repression of the cGAS–STING signaling axis. Promoter hypermethylation of cGAS and STING has been associated with transcriptional repression, attenuated type I IFN signaling, limited MHC class I upregulation, and reduced T cell recognition in melanoma models (23). Pharmacological demethylation restored STING signaling and enhanced tumor recognition in preclinical experimental systems. These findings indicate potential biological reversibility but do not establish therapeutic actionability in patients. The effects of demethylating agents are genome-wide and are not restricted to the cGAS or STING promoters. Candidate readouts may include cGAS and STING expression, locus-specific methylation, downstream type I IFN signaling, and functional responses to STING activation. However, cGAS–STING methylation status has not been prospectively validated as a predictive biomarker or as a criterion for selecting patients for demethylating treatment. Therefore, the proposed innate immune-silenced state remains a preclinical mechanistic construct rather than a clinically actionable category. Although cGAS–STING promoter methylation may provide a mechanistically informative candidate biomarker for impaired innate immune sensing, its therapeutic relevance is unclear. Current demethylating agents act broadly across the genome and are not selective for the cGAS–STING axis or for melanoma cells. Therefore, cGAS–STING methylation status should not currently be considered an actionable biomarker or a validated criterion for selecting patients for epigenetic priming. Prospective studies integrating locus-specific methylation, pathway activity, cell-type-resolved analyses, and clinical outcomes will be required to determine whether this feature has predictive or pharmacodynamic value.

#### 5.3.3 Dedifferentiated immune-adaptive state

A third proposed configuration corresponds to a dedifferentiated immune adaptive phenotype, closely linked to melanoma phenotype switching and transcriptional plasticity. These tumors are typically defined by MITF low/AXL high programs, reduced melanocytic differentiation, inflammatory signaling activation, and increased invasiveness (53, 55). As discussed in the context of phenotype switching, inflammatory cytokines and therapeutic pressure can induce extensive chromatin-remodeling programs that favor dedifferentiation, altered antigenicity, and adaptation to immune selection. Nevertheless, targeted-therapy-induced drug-tolerant states, inflammation-induced loss of melanocytic antigens, and invasive WNT5A-associated phenotypes arise from distinct experimental contexts and should not be considered interchangeable manifestations of a single state. In an experimental model of adoptive T cell therapy, sustained TNF-α signaling induced reversible loss of melanocytic differentiation antigens and reduced recognition by antigen-specific T cells (57). During RAF/MEK inhibition, single-cell analyses identified multiple coexisting drug-tolerant transcriptional states (51). Collectively, these observations support an association between melanoma cell plasticity, therapeutic pressure, and altered immune recognition, but do not establish the MITF-low/AXL-high profile as a validated predictor of ICI resistance.

#### 5.3.4 IFN-responsive but immune-exhausted state

Some melanomas display preserved inflammatory signaling yet remain therapeutically resistant. These tumors may exhibit an IFN-responsive but T-cell dysfunctional configuration, characterized by sustained IFN signaling, inflammatory transcriptional programs, and increased PD-L1 expression, accompanied by dysfunctional or exhausted T cell populations (21, 46, 75). This proposed state spans at least two biological compartments: IFN-responsive melanoma cells and infiltrating T cells with variable functional capacity. Chronic inflammatory signaling and checkpoint expression do not, by themselves, establish terminal T cell exhaustion. Conventional single-parameter readouts such as an IFN-γ signature or PD-L1 positivity alone would classify these tumors as ICI-sensitive, whereas their defining feature is the disconnection of inflammatory signaling from functional cytotoxicity.

Assessment must differentiate progenitor-like or stem-like dysfunctional T cells, often linked to TCF7 expression, from terminally exhausted populations defined by various combinations of inhibitory receptors, transcription factors, proliferative capacity, and cytotoxic function. Consequently, TCF7 should not be categorized merely as an exhaustion marker.

Candidate measurements may encompass markers associated with terminal T cell dysfunction or exhaustion, including Thymocyte selection-associated high mobility group box (TOX), T cell immunoglobulin and mucin-domain containing-3 (TIM-3), Lymphocyte Activation Gene-3 (LAG-3), Transcription factor-7 (TCF7)may instead help identify progemitor-like or stem-like dysfunctional T cell populations. No standardized marker panel or threshold currently defines this state or prospectively predicts ICI response in melanoma. Its characterization therefore requires integrated tumor cell, immune-phenotypic, spatial, and functional analyses rather than inference from PD-L1 or an IFN signature alone (75, 76).

#### 5.3.5 Myeloid-dominant immunosuppressive state

In addition to tumor intrinsic programs, the composition of the tumor microenvironment can establish a myeloid dominant, immunosuppressive state characterized by an accumulation of macrophages and MDSCs, along with regulatory cytokine networks and diminished infiltration of CD8+ T cells. Suggesting that epigenetic regulators may affect the differentiation and function of suppressive myeloid populations. Nonetheless, melanoma-specific evidence directly linking a specific myeloid chromatin state to ICI resistance is still scarce. (48) identifies a correlation between melanoma HDAC4 expression and reduced T cell inflamed signatures, yet it does not directly demonstrate the epigenetic maintenance of macrophage or MDSC programs. It is therefore insufficient on its own to validate a myeloid-dominant epigenetic state (47). This proposed configuration is primarily microenvironmental rather than tumor intrinsic and it may provide a candidate framework for characterizing the immunosuppressive organization of the TIME. However, its value for patient stratification must be established through prospective clinical validation. Composite signatures, including myeloid-to-CD8^+^ ratios, suppressive cytokine profiles, and chromatin-state readouts, more effectively encapsulate this condition than any individual marker. Bulk tumor analyses are insufficient to differentiate tumor cell epigenetic programs from those of infiltrating myeloid cells without cellular enrichment, computational deconvolution, single-cell profiling, or spatial analysis. Although this configuration may justify combinations with myeloid-directed or epigenetic agents, it does not presently identify patients who have clinically demonstrated benefits from HDAC inhibition, macrophage-targeting strategies, or innate immune modulation (77).

#### 5.3.6 Epigenetically reactivated antigenic state

Finally, therapeutic intervention itself may induce an epigenetically reactivated antigenic state, particularly following DNA methyltransferase inhibition. DNMT inhibitors can restore expression of CTAs, including MAGE-A, NY-ESO-1, GAGE, and SSX, thereby increasing tumor antigenicity and potentially enhancing responsiveness to checkpoint blockade or vaccine-based immunotherapies (37–39, 61). Unlike earlier stages, this condition is not an intrinsic tumor trait, but rather an inducible phenotype. This demonstrates how epigenetic therapies can reshape melanoma immunogenicity, creating periods of immune vulnerability that are useful for treatment. In this context, TA re-expression may serve as a candidate pharmacodynamic indicator of target engagement; however, it should not be treated as a validated predictive biomarker for sensitivity to subsequent or concurrent immunotherapy. Increased CTA expression does not necessarily result in effective antigen processing, presentation, or T cell recognition. Longitudinal monitoring of CTA expression or methylation may be useful in research studies examining the timing and biological effects of epigenetic priming. However, no clinically validated expression threshold or sampling schedule currently defines an optimal therapeutic window for combining DNMT inhibition with ICIs. Clinical studies evaluating epigenetic agents combined with ICIs in melanoma, together with relevant cross-tumor evidence, are summarized in **Table 2**.

**Table 2:** Selected clinical studies evaluating epigenetic agents combined with immune checkpoint inhibitors in melanoma and relevant cross-tumor evidence.

Study and disease scope	Treatment and design	Population	Efficacy findings	Safety	Publication status	Principal limitations
NIBIT-M4 NCT02608437 Melanoma-specific	Guadecitabine plus ipilimumab; phase Ib, single-center, open-label, dose-escalation (3 + 3)	19 patients with unresectable stage III/IV melanoma; 18 treatment-naive and 1 previously treated with anti-PD-1	irORR 26%; irDCR 42%. At long-term follow-up: median PFS 5.2 months, median OS 25.6 months, median DoR 20.6 months, and 5-year OS 28.9%.	Grade 3/4 adverse events in 84%; no protocol-defined dose-limiting or overlapping toxicities were reported.	Peer-reviewed primary report and 5-year follow-up with exploratory multi-omic analysis Di Giacomo et al., (78); Noviello et al., (79)	Very small, non-randomized cohort; no ipilimumab-alone control; primarily treatment-naive population; efficacy contribution of guadecitabine cannot be isolated; exploratory correlative analyses involved smaller sample subsets.
ENCORE-601 melanoma cohort Anti-PD-1-refractory melanoma	Entinostat plus pembrolizumab; phase Ib/II, multicenter, open-label, single-arm expansion cohort	53 evaluable patients with melanoma progressing during or after prior anti-PD-1 therapy	10 objective responses (1 CR and 9 PR); ORR 19%. Median DoR 12.5 months at the reported cutoff; 5 responses were ongoing.	Conference reports described grade ≥3 treatment-related adverse events in 14 patients; 6 discontinued because of treatment-related adverse events, including immune-mediated events.	Conference abstracts/meeting reports; no full peer-reviewed melanoma-specific trial publication identified Johnson et al., (63); Sullivan et al., (70)	Single-arm design; modest sample size; no pembrolizumab rechallenge control; selected anti-PD-1-experienced population; conference-level reporting; activity cannot be attributed specifically to HDAC inhibition.
ENCORE-601 NSCLC cohort Cross-tumor evidence	Entinostat plus pembrolizumab; phase Ib/II, open-label, single-arm study	76 treated patients with metastatic NSCLC previously exposed to anti-PD-(L)1; 71 evaluable for efficacy	irRECIST ORR 9.2% (95% CI 3.8-18.1); median DoR 10.1 months and median OS 11.7 months. The prespecified response-rate threshold was not met.	The combination had a manageable but clinically relevant toxicity profile; treatment-related adverse events and discontinuations occurred.	Full peer-reviewed publication Hellmann et al., (64)	Different tumor type; cannot validate epigenetic priming in melanoma; single-arm design; primary response endpoint not met; any comparison with melanoma is indirect.

Interpretation: The melanoma-specific evidence remains early-stage and does not establish that epigenetic therapy restores sensitivity to immune checkpoint blockade. Cross-tumor findings are included only as indirect context and should not be interpreted as melanoma-specific validation (63, 64, 70, 78, 79). CI, confidence interval; CR, complete response; DoR, duration of response; HDAC, histone deacetylase; ICI, immune checkpoint inhibitor; irDCR, immune-related disease-control rate; irORR, immune-related objective response rate; irRECIST, immune-related Response Evaluation Criteria in Solid Tumors; NSCLC, non-small-cell lung cancer; ORR, objective response rate; OS, overall survival; PFS, progression-free survival; PR, partial response.

#### 5.3.7 Validation requirements for the proposed framework

The proposed states are not mutually exclusive. A melanoma lesion may simultaneously display impaired antigen presentation, attenuated innate sensing, a dedifferentiated tumor cell phenotype, dysfunctional T cells, and a myeloid-dominant microenvironment. Conversely, distinct metastatic lesions from the same patient may show different configurations. Longitudinal changes may reflect true state transitions, altered cellular composition, spatial sampling, or selection of pre-existing subpopulations. Advancing this framework requires longitudinal molecular profiling of clinically annotated specimens. Serial samples obtained prior to treatment, early in therapy, at the point of response, and at progression could help in differentiating between enduring resistance mechanisms and treatment-induced adaptations. When repeated tissue biopsies are not feasible, circulating tumor DNA, blood immune profiling, or other minimally invasive samples may offer supplementary insights, yet they cannot entirely replicate the spatial and cellular data inherent in tumor tissue (80).

## 6 Biobanking and integrative multi-omics: a framework for biomarker discovery in melanoma

The significant clinical heterogeneity of melanoma, particularly in its response to ICIs and targeted therapies, highlights the urgent need for biomarkers that can predict therapeutic benefit, resistance, and disease progression. However, the immune response in melanoma cannot be adequately explained by a single molecular layer or static biomarker; rather, therapeutic response emerges from the dynamic interplay of genomic alterations, epigenetic plasticity, immune pressure, and TIME remodeling. A major research challenge is differentiating persistent resistance mechanisms from treatment-related adaptations and ascertaining which of these adaptations are functionally reversible. In this context, the translational value of biobanking is not limited to the storage of biological samples. In this regard, longitudinal biobanking integrated with multi-omic profiling provides a uniquely powerful framework for capturing the temporal evolution of tumor immune interactions under therapeutic pressure. This enables researchers to distinguish between stable genomic resistance mechanisms and reversible, epigenetically driven immune evasion states to identify patients who are most likely to benefit from epigenetic priming strategies and effective immunotherapy combinations (24). However, longitudinal molecular changes alone do not demonstrate epigenetic causality or reversibility, nor do they identify patients who will benefit from epigenetic priming. Such interpretations require cell-type-resolved analyses, functional validation, and prospective clinical assessment. A conceptual overview of this proposed biobank-enabled research framework is presented in **Figure 2**.

**Figure 2:**
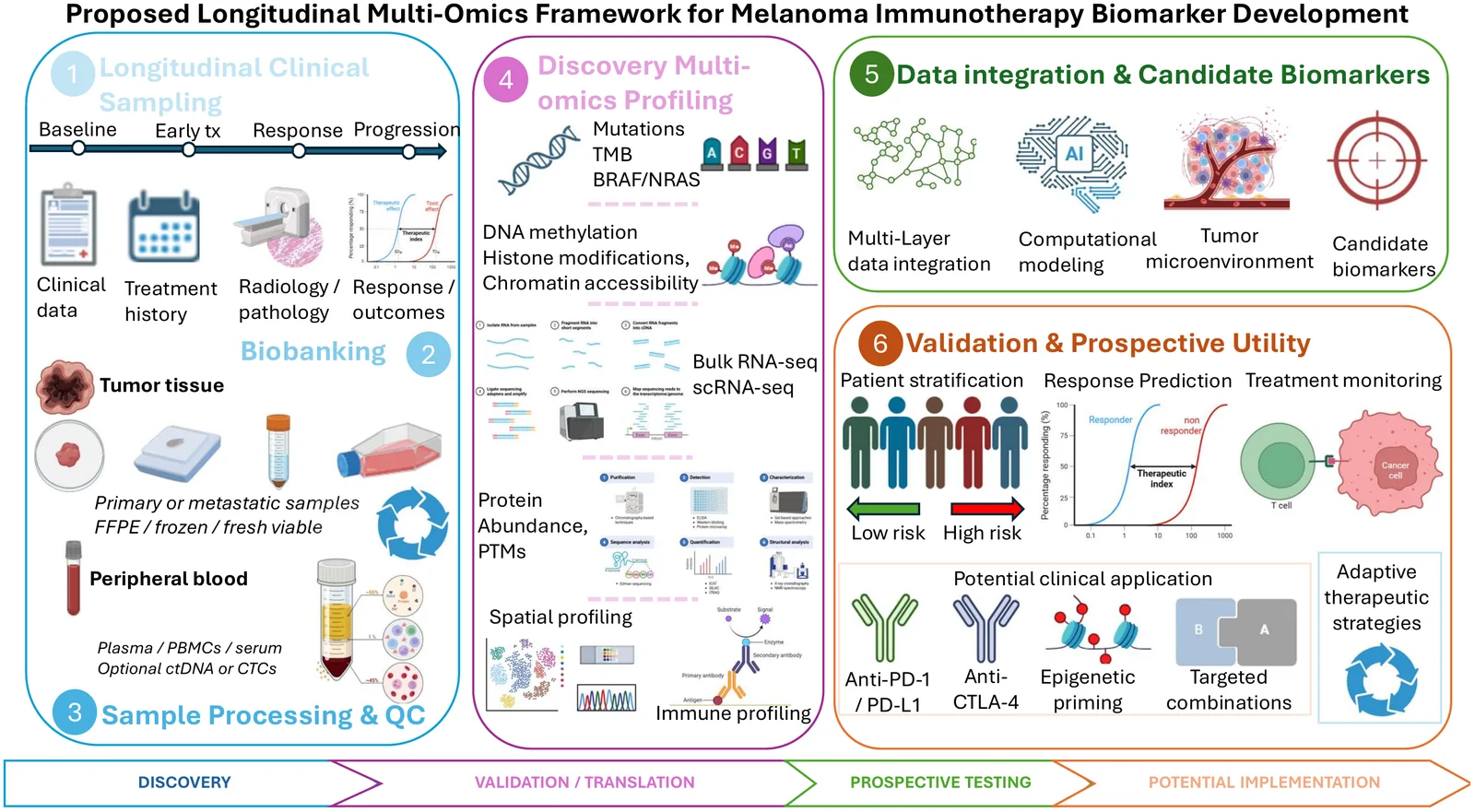
Longitudinal multi-omics framework for melanoma immunotherapy biomarker development. The figure presents a proposed research framework linking longitudinal clinical sampling and biobanking to multi-omics discovery, candidate biomarker development, validation and prospective assessment of clinical utility. (1) Longitudinal Clinical Sampling: clinical data, treatment history, radiological/pathological assessments and response/outcome information are collected across clinically relevant time points, including baseline, early treatment, response and progression. (2) Biobanking: longitudinal biospecimens include primary or metastatic tumor tissue collected as FFPE, frozen, or fresh viable material, together with peripheral-blood derivatives such as plasma, PBMCs and serum; ctDNA and CTCs may be assessed where appropriate. (3) Sample Processing & QC: sample processing and quality control are intended to minimize pre-analytical variability and support reproducibility across longitudinal collections and participating centers. (4) Discovery Multi-omics Profiling: biospecimens undergo integrated genomic, epigenomic, transcriptomic, proteomic, spatial and immune profiling to characterize tumor intrinsic and microenvironmental biology. (5) Data Integration & Candidate Biomarkers: multi-layer molecular data are integrated using computational and statistical/machine-learning approaches to characterize the tumor microenvironment and generate candidate biomarker signatures. These signatures represent discovery-stage hypotheses rather than clinically validated predictive tools. (6) Validation & Prospective Utility: candidate biomarkers require validation and translation before prospective assessment of potential applications such as patient stratification, response prediction, treatment monitoring and biomarker-informed therapeutic strategies. The framework explicitly distinguishes discovery, validation/translation, prospective testing and potential future implementation; clinical application requires reproducible, clinically compatible assays and prospective demonstration of utility. ctDNA, circulating tumor DNA; CTC, circulating tumor cell; FFPE, formalin-fixed paraffin-embedded; PBMC, peripheral blood mononuclear cell; PTM, post-translational modification; QC, quality control; RNA-seq, RNA sequencing; scRNA-seq, single-cell RNA sequencing; TMB, tumor mutational burden; PD-1, programmed cell death protein 1; PD-L1, programmed death-ligand 1; CTLA-4, cytotoxic T-lymphocyte-associated protein 4. Figure created with BioRender.com.

Importantly, specimens collected over time and annotated with clinical data enable us to characterize the evolution of melanoma across disease progression and treatment exposure. This approach can support investigation of dynamic changes in tumor plasticity, immune remodeling, and therapy-induced molecular adaptation. Through serial sampling of tumor tissue, matched blood specimens, and metastatic lesions, one may investigate tumor evolutionary trajectories and discover candidate molecular features associated with treatment sensitivity or resistance (81). An informative sampling strategy should include pretreatment baseline, early on-treatment assessment, response or persistent disease, and progression, with documentation of treatment exposure, biopsy timing, and anatomical site. When feasible, sampling of different lesions may help assess intermetastatic heterogeneity.

Repeated tissue biopsies are nevertheless constrained by accessibility, procedural risk, patient burden, and tissue availability. Matched blood specimens, including plasma for circulating tumor DNA and peripheral immune-cell profiling, can provide complementary longitudinal information but do not fully reproduce the cellular and spatial features of tumor tissue. Furthermore, apparent temporal changes may reflect differences in biopsy location or cellular composition rather than true transitions within the same tumor cell population.

In this context, biobanks are evolving from passive repositories of biological material into active translational infrastructures that enable integrative, systems-level interrogation of melanoma biology. Standardized biospecimen collection and processing are essential for omics applications since pre-analytical variability can significantly impact molecular profiling, reproducibility, and biological interpretation (24, 82).

Identifying molecular determinants of immunotherapy response requires integrative analyses of high-quality, clinically annotated patient specimens. Epigenomic, transcriptomic, proteomic, immune, and spatial profiling can provide complementary information on tumor intrinsic and microenvironmental biology. However, the inclusion of additional molecular layers does not necessarily improve predictive performance or clinical utility, and their added value should be evaluated against simpler and more reproducible assays. This multidimensional strategy may support the investigation and future validation of the potentially overlapping epigenetic immune configurations described in **Table 1**, including antigen-presentation-deficient, innate immune-silenced, dedifferentiated immune-adaptive, IFN-responsive but T-cell dysfunctional, myeloid-dominant suppressive, and treatment-induced antigen-reactivation configurations. These proposed configurations should not be interpreted as discrete or clinically validated categories. Distinguishing tumor cell intrinsic programs from immune or stromal signals will require cellular enrichment, validated computational deconvolution, single-cell profiling, or spatial approaches, particularly when interpreting bulk methylation or transcriptomic data. Computational and machine-learning approaches may support the integration of multidimensional longitudinal datasets and the identification of candidate biomarker combinations. Their development, however, requires adequate sample sizes, prespecified clinical endpoints, appropriate feature reduction and missing-data handling, prevention of data leakage, control of batch and center effects, rigorous internal validation, and independent external validation. Preliminary conference data from spatial multi-omic profiling of mucosal melanoma suggest that spatially organized tumor–immune features may differ between responders and non-responders to immune checkpoint blockade. In this study, response-associated samples contained spatial immune-engaged niches involving proliferating tumor cells, macrophages, and dendritic cells, whereas resistant samples exhibited stromal features associated with immune exclusion. These preliminary observations suggest that spatial organization may provide information beyond cellular composition alone. However, the findings derive from conference-reported data and require validation in larger, independent cohorts before they can be considered clinically predictive (83).

In summary, the integration of biobanking and multi-omics technologies provides a research infrastructure for studying melanoma heterogeneity and the molecular correlates of therapeutic response and resistance. Translation requires a staged progression from discovery to analytical validation, independent clinical validation, and prospective demonstration of clinical utility. Candidate multi-omics signatures must ultimately be reduced to reproducible assays with defined thresholds, feasible turnaround times, and evidence that their use improves clinical decisions or outcomes. Thus, clinically annotated longitudinal biobanks may support future biomarker development and validation, but neither biobanking nor multi-omics integration alone establishes clinically actionable precision immunotherapy.

## 7 Future perspectives

Melanoma provides a valuable model for investigating biological adaptation, in which therapeutic response reflects the evolving relationship between tumor progression and immune control. Shifting from a static view of tumor immune interactions to a more flexible one could transform precision oncology, turning treatment from a reactive process into one guided by the disease’s evolution. In this context, spatial epigenomics will likely become an essential tool because it enables the study of epigenetic states within their native tissue structure, revealing spatially organized mechanisms of immune escape, cellular plasticity, and therapeutic resistance that remain hidden in bulk or single-cell analyses. Thus, future progress may rely less on isolated biomarkers and more on interpreting dynamic biological changes across time and space. This may support the development of more adaptive and mechanistically informed therapeutic strategies.

## 8 Conclusions

In summary, melanoma is a model in which tumor evolution, immune resistance, and therapeutic response emerge from an evolving interplay of genomic alterations, epigenetic flexibility, and environmental influences. Although immune checkpoint inhibitors have greatly improved melanoma treatment, their long-term efficacy is limited by adaptive resistance mechanisms that may be influenced, in part, by epigenetic regulation. Here, we summarize evidence indicating that DNA methylation, histone modifications, and chromatin remodeling may contribute to immune-evasive programs at multiple levels and interfere with antigen presentation, innate immune sensing, T cell function, and tumor adaptability. However, the reversible nature of these processes makes epigenetic alterations a source of therapeutic resistance and a potential target for re-sensitizing melanoma to immunotherapy. This review brings together current evidence and outlines an integrative framework in which biobanking resources and multilayer multi-omics profiling are important tools to understanding the molecular factors that shape immunotherapy response and resistance. Combining epigenomic, transcriptomic, spatial, and immune data may support the transition from static biomarkers toward more dynamic and biologically informed patient classification. The intersection of epigenetic therapies, immune checkpoint blockade, and biobank-based systems biology may contribute to the future development of biomarker-informed therapeutic strategies in melanoma. This integration could support the future evaluation of adaptive therapeutic strategies suited to changes in the tumor immune environment.
